# A Delphi Process to Optimize Quality and Performance of Drug Evaluation in Neonates

**DOI:** 10.1371/journal.pone.0104976

**Published:** 2014-09-11

**Authors:** Frederic Legrand, Rym Boulkedid, Valery Elie, Stephanie Leroux, Elizabeth Valls, Adolfo Valls-i-Soler, Johannes N. Van den Anker, Evelyne Jacqz-Aigrain

**Affiliations:** 1 AP-HP (Assistance Publique des Hôpitaux de Paris), Department of Paediatric Pharmacology and Pharmacogenetics, Inserm CIC 9202 (Centre d'Investigation Clinique pédiatrique), University Diderot Paris VII, Hôpital Robert Debré, Paris, France; 2 AP-HP (Assistance Publique des Hôpitaux de Paris), Unité de Recherche Clinique (URC), Inserm CIE5 (Centre d'Investigation clinique Epidémiologie clinique), Hôpital Robert Debré, Paris, France; 3 University of Basque country, Head Neonatal Intensive care Unit, Cruces University Hospital, Barakaldo-Bilbao, Spain; 4 Evan and Cindy Jones Professor of Paediatric Clinical Pharmacology Chief, Division of Clinical Pharmacology, Children's National Health System, Washington, District of Columbia, United States of America; Fondazione IRCCS Policlinico San Matteo, Italy

## Abstract

**Background:**

Neonatal trials remain difficult to conduct for several reasons: in particular the need for study sites to have an existing infrastructure in place, with trained investigators and validated quality procedures to ensure good clinical, laboratory practices and a respect for high ethical standards. The objective of this work was to identify the major criteria considered necessary for selecting neonatal intensive care units that are able to perform drug evaluations competently.

**Methodology and Main Findings:**

This Delphi process was conducted with an international multidisciplinary panel of 25 experts from 13 countries, selected to be part of two committees (a scientific committee and an expert committee), in order to validate criteria required to perform drug evaluation in neonates. Eighty six items were initially selected and classified under 7 headings: “NICUs description - Level of care” (21), “Ability to perform drug trials: NICU organization and processes (15), “Research Experience” (12), “Scientific competencies and area of expertise” (8), “Quality Management” (16), “Training and educational capacity” (8) and “Public involvement” (6). Sixty-one items were retained and headings were rearranged after the first round, 34 were selected after the second round. A third round was required to validate 13 additional items. The final set includes 47 items divided under 5 headings.

**Conclusion:**

A set of 47 relevant criteria will help to NICUs that want to implement, conduct or participate in drug trials within a neonatal network identify important issues to be aware of.

**Summary Points:**

1) Neonatal trials remain difficult to conduct for several reasons: in particular the need for study sites to have an existing infrastructure in place, with trained investigators and validated quality procedures to ensure good clinical, laboratory practices and a respect for high ethical standards. 2) The present Delphi study was conducted with an international multidisciplinary panel of 25 experts from 13 countries and aims to identify the major criteria considered necessary for selecting neonatal intensive care units (NICUs) that are able to perform drug evaluations competently. 3) Of the 86 items initially selected and classified under 7 headings - “NICUs description - Level of care” (21), “Ability to perform drug trials: NICU organization and processes (15), “Research Experience” (12), “Scientific competencies and area of expertise” (8), “Quality Management” (16), “Training and educational capacity” (8) and “Public involvement” (6) - 47 items were selected following a three rounds Delphi process. 4) The present consensus will help NICUs to implement, conduct or participate in drug trials within a neonatal network.

## Introduction

In the neonatal population, more than 90% of products are used unauthorised or off-label, especially when neonates are treated in Neonatal Intensive Care Units (NICUs). New treatments are often introduced in the neonatal therapeutic arena without specific evaluation, on the basis that they have proven efficacy in adults and older children. Altogether, many drugs, even when authorised for use in neonates, would benefit from further validated data and a consensus among neonatologists to ensure the most optimal use. In order to improve this situation, a few initiatives were undertaken starting in the USA with the Food and Drug Administration Modernization Act and Best Pharmaceuticals for Children Act respectively in 1997 and 2002, and the American Paediatric Research Equity Act and Newborn Drug Initiative respectively in 2003 and 2006. Similarly in Europe, the European Paediatric Regulation entered in force in June 2007 to increase the development of medicines for all paediatric age groups, including neonates.

There are many major and already well-known practical and ethical issues in conducting drug evaluation in neonates [1], [2]. Although neonates represent only a small part of the population, they have specific diseases and high variations in disease presentations with a major risk of unfavorable long-term outcome. Evaluation of drug pharmacokinetics, efficacy and safety are required in the different neonatal age groups from 24 to 44 weeks' gestational age, characterised by differences in physiological and pharmacological maturation affecting drug disposition and effects [3], [4]. Suitable approaches, adapted to neonates are recommended but need to be more widely used: population pharmacokinetic and bridging studies [5], [6], adapted designs and other methodologies recognised as pertinent to evaluate efficacy when randomized controlled trials are not possible [7]–[9].

Many of these issues can be solved by bringing together scattered expertise within a neonatal network dedicated to drug evaluation in neonates, in collaboration with the European Society of Developmental, Perinatal and Paediatric Pharmacology (ESDPPP). According to the request of the European Commission and following the EnprEMA network initiative (European Network of Paediatric Research at the European Medicines Agency), we conducted a Delphi process [10]–[14] to identify the criteria that would help neonatologists to organise a NICU research infrastructure in order to conduct drug evaluation trials and be part of a European network for drug evaluation in neonates.

## Methods

### Ethics Statement

The study did not need institutional review board approval as it did not affect patient care, and the information that it generated was used for consensual quality criteria only.

The objectives of the Delphi study were presented to all participants, their agreement and availability to participate were obtained (their written consent consisted of replying positively by email to the invitation sent by the organisers), and their independency verified.

### Organization of the Delphi Study

The objective of the Delphi study was to obtain a consensus on the prerequisites that NICUs need to fulfil in order to perform clinical drug trials. Two experts groups conducted the Delphi study by email between February 2012 and February 2013.

### Composition of the Scientific and Expert Committees

Two expert groups conducted the Delphi study: the Scientific Committee consisted of 10 experts responsible for organising the Delphi device, selecting and/or approving the members of the Expert Committee, drafting the successive versions of the questionnaire, and analysing the data after each round (questions and answers, results). All members of the Expert Committee (the panellists) agreed to answer the questionnaire during each round and to criticize the answers if necessary.

All the participants (**Table 1**) were initially selected by the organisers (FL & EJA) in order to ensure that they will represent all potential differences in background, occupational environment, clinical approaches or practices. They were contacted because of their recognised expertise in the different areas of knowledge required for drug evaluation in neonates (neonatology, pharmacology, clinical research), after evaluation of teaching functions, publications, participations to international scientific societies, scientific and ethic boards or networks). The composition of the Scientific and Expert committees included clinical investigators (paediatricians or neonatologists), pharmacologists or pharmacists, researchers, regulators, and employed by industry, academia or regulatory agencies and in most cases members of scientific societies and clinical networks. They were from various countries/continents, had a broad range of ages and different levels of expertise. All the members of the Scientific committee agreed on the composition of the groups, and accepted to participate. The expert selection was then submitted for approval to the Scientific Committee, and finally two additional members were added upon their suggestions.

### Questionnaire preparation

The Scientific Committee drafted the first version of the questionnaire, composed of questions and simple items. Each member proposed recognition criteria and a total of 105 items were listed. They were then invited to confirm and/or refine all the items to allow them to be rated during the Delphi process. The resulting questionnaire was submitted a third time by the organisers, for validation by each member of the Scientific Committee. Finally, the elaborated questionnaire included 86 items divided over 7 headings: (**Table 2** **, column A**): H1: “NICUs description - Level of care” (21 items), H2: “Ability to perform drug trials: NICU organization and processes” (15 items), H3: “Research experience of the NICU” (12 items), H4: “Scientific competencies and capacity to provide expert advice” (8 items), H5: “Quality management” (16 items), H6: “Training and educational capacity” (8 items), and H7: “Public involvement” (6 items).

### Rounds

As required in a Delphi study, each item is required to be assessed twice, using a 1^st^ and a 2^nd^ level of consensus to be finally selected.

### First round

The first round was performed from February to July 2012. The panellists (Expert Committee) received the first questionnaire by electronic mail and they were invited to rate their agreement about each item on a 9-point scale, where 1 meant definitely not agree and 9 definitely agree. They were also invited to comment on each item using a dedicated “comment box”, and/or to add items considered as important. Items were included in the second round if a consensus was reached based on two selection criteria: a median score in the top tertile (7–9) and at least 65% of panel ratings in the top tertile (1^st^ level of consensus). At the end of the first round, the questionnaire was slightly modified by the scientific committee and a few items were added to take into account comments and suggestions of the panellists.

### Second round/Third round

The second round was conducted between November and December 2012. All panellists who had participated in the first round were sent the second-round questionnaire by email, with the results of the first round including median panel rating, frequency distribution rating as well as their individual ratings from the first round. They were asked to re-score each item based on their own opinion, and the panel responses obtained during the first round. The second round also included a limited number of “new” items, not evaluated in the first round and that had to send out from a second evaluation during what has been called a “third round” in order to evaluate each item twice. This third round took place between January and February 2013. To be included in the final list, the items were selected by the level of median rating in the top tertile (7–9) and a 75% agreement among panellists that the rating was in the top tertile (7–9) (2^nd^ level of consensus).

## Results

The characteristics of the two experts groups (n = 25) are presented **Table 1** and they are all listed in alphabetic order in the “Acknowledgment” section. They were selected because they had at least 10 years' experience and a well-known international recognition in their field of expertise.

**Table 1:** Main characteristics of the Expert and Scientific Committees.

CHARACTERISTICS	Scientific Committee (n = 10)	Expert Committee (n = 15
Sex, n (%)		
Female	2 (20)	3 (20)
Male	8 (80)	12 (80)
Age (years), median (q1, q3)	51 (44, 58)	52 (49, 57)
Years of experience, median (q1, q3)	22 (19, 29)	24 (21, 30)
Present professional setting, n (%)		
Industrial/Private	0 (0)	2 (13)
Institutional	10 (100)	13 (87)
Speciality, n (%)		
Regulation and Trial management	1 (10)	4 (27)
Pediatric pharmacology	3 (30)	2 (13)
Neonatology	6 (60)	9 (60)
Geographical origin, n (%)		
Europe	6.5 (65)	11 (73)
Asia	0 (0)	2 (13)
US/Canada	2.5 (25)	1 (7)
Australia	1 (10)	1 (7)

**Table 2:** Results of the Delphi process.

BOLD ITEMS MEET THE TWO SELECTION CRITERIA OF THE 1^st^ and the 2^nd^ CONSENSUS LEVELS (i.e. 1^st^ LEVEL: MEDIAN SCORE ≥ 7 and PERCENT AGREEMENT WITH {7 ≤ SCORE ≤ 9} ≥ 65%; and 2^nd^ LEVEL: MEDIAN SCORE ≥ 7 and PERCENT AGREEMENT WITH {7 ≤ SCORE ≤ 9} ≥ 75%)										
“COLUMN A” - INITIAL SUGGESTED ITEMS		“COLUMN B” - MODIFIED ITEMS AND/OR HEADINGS AFTER THE 1^ST^ ROUND		1^st^ ROUND		2^nd^ ROUND		3^rd^ ROUND		FINAL SELECTED ITEMS
1. NICUs DESCRIPTION - LEVEL OF CARE		1. NICUs DESCRIPTION		MEDIAN	% AGREEMENT (7–9)	MEDIAN	% AGREEMENT (7–9)	MEDIAN	% AGREEMENT (7–9)	
		1.1. LEVEL OF CARE OF THE NICU								
1	Number of staff members			7	73,3	7	73,3			
2	Number of beds available for research			7	66,7	7	73,3			
**3**	**Number of doctors/bed**			**7**	**66,7**	**7**	**80,0**			**✓**
**4**	**Number of nurses/bed**			**7**	**73,3**	**7**	**80,0**			**✓**
5	Possibility to keep kids a day more in the NICU for research purposes			6	46,7					
**6**	**Ventilation management with mechanical ventilation support is available**	**Ventilation management with a range of respiratory support available (including CPAP and mechanical ventilation)**		**8**	**86,7**	**8**	**93,3**			**✓**
7	Inhaled nitric oxide is available (6')	6′	List of specialized care technics available: inhaled nitric oxide, ECMO, neonatal surgery, body cooling…	6	46,7	7	64,3 (<65%)			
8	ECMO is available (6')			3	13,3					
9	Body cooling is available (6')			5	40,0					
10	Neonatal surgery is available in the hospital (6')			7	60,0					
11	Patient transfert to special center is available			7	53,3					
**12**	**Patient follow-up after discharge is available**			**8**	**100,0**	**9**	**100**			**✓**
13	Annual report on medical activities			8	69,2	7	73,3			
**14**	**Number of infants yearly admitted**			**8**	**93,3**	**8**	**93,3**			**✓**
**15**	**Number of patients <1500 gram (VLBW) birth weight yearly admitted**			**7**	**86,7**	**7**	**93,3**			**✓**
**16**	**Number of patients <1000 gram (ELBW) birth weight yearly admitted**			**7**	**80,0**	**7**	**80,0**			**✓**
17	Number of surgical infants yearly admitted			6	33,3					
18	Number of readmissions infants yearly admitted			4	21,4					
19	Number of ventilated patients per year			7	60,0					
20	Number of kids inborn			7	66,7	7	66,7			
21	Number of kids outborn			5	26,7					

3. RESEARCH EXPERIENCE OF THE NICU		1. NICUs DESCRIPTION	MEDIAN	% AGREEMENT (7–9)	MEDIAN	% AGREEMENT (7–9)	MEDIAN	% AGREEMENT (7–9)	FINAL SELECTED ITEMS
		1.2. NICU RESEARCH EXPERIENCE IN DRUG TRIALS: This heading includes items required: 1 - to evaluate once yearly Established units (NICUs with experiences that have already conducted and/or participated in clinical trials). These units will do well on these criteria; 2 - to evaluate Development units (NICUs recently or not yet involved that have the capacity to do research but don't have a track record. NOT REQUIRED TO ENTER THE NETWORK, but for the regular annual assessment.							
**37**	**Type of clinical trials performed over the last 5 years (phase I/II, phase III/IV, observational studies, PK, PD, PK/PD, efficacy, safety, pharmacoepidemiologic, pharmacovigilance, diagnostic and comportment study, follow-up, etc…)**		**7**	**73,3**	**7**	**69,2**	**7,5**	**85,7**	**✓**
**38**	**Number of completed and ongoing clinical trials over the last 5 years (single centre, national multicenter and international multicenter trials)**		**7**	**66,7**	**8**	**78,6**	**8**	**92,9**	**✓**
**39**	**Number of investigator (academic) initiated studies performed**		**7**	**53,3**	**7**	**71,4**	**7**	**78,6**	**✓**
40	Proportion of academic or industrial sponsored studies	Number of industrial sponsored studies	6	46,7	7	57,1 (<65%)			
41	Number of principal investigators in clinical trials		7	66,7	6,5	50,0 (<65%)			
**42**	**Numbers of patients enrolled in these trials**		**7**	**60,0**	**7**	**71,4**	**7,5**	**92,9**	**✓**
43	Numbers of PhD trainees		5	26,7					
44	Numbers of peer reviewed publications		7	60,0					
**45**	**Success rate of real enrollment of the study**		**8**	**80,0**	**8**	**85,7**	**8**	**85,7**	**✓**
**46**	**Success rate of conduct of the study (adherence to timelines)**		**8**	**80,0**	**8**	**92,3**	**8**	**100**	**✓**
**47**	**Taking part as a member in neonatal/paediatric networks**		**8**	**73,3**	**7**	**78,6**	**8**	**100**	**✓**
48	Annual report of research activities		7	53,3	7	64,3 (<65%)			

6. TRAINING AND EDUCATIONAL CAPACITY		1. NICUs DESCRIPTION		MEDIAN	% AGREEMENT (7–9)	MEDIAN	% AGREEMENT (7–9)	MEDIAN	% AGREEMENT (7–9)	FINAL SELECTED ITEMS
		1.3. TRAINING AND EDUCATIONAL CAPACITY OF THE NICU								
**73**	**Internal training regarding GCPs and human subjects (research ethics) is available**			**7,5**	**78,6**	**7**	**93,3**			**✓**
**74**	**Internal training regarding responsibilities of principal investigator and all co-investigators is available**			**8**	**78,6**	**8**	**100**			**✓**
**75**	**Training courses specific to planned trial**			**8**	**85,7**	**8**	**80**			**✓**
76	Training opened to external participant			7	53,3					
77	Team involved in external training (75')	75′	Team involved in external national and international training courses	7	53,3	7	60,0 (<65%)			
78	Participation to national or international training courses (75')			7,5	64,3					
79	Kind of internal/external courses taken			6,5	50,0					
80	Kind of internal/external courses given			6	35,7					

2. ABILITY TO PERFORM DRUGS TRIALS: NICU ORGANISATION AND PROCESSES		2. NICU ORGANISATION AND PROCESSES (ABILITY TO PERFORM DRUGS TRIALS)		MEDIAN	% AGREEMENT (7–9)	MEDIAN	% AGREEMENT (7–9)	MEDIAN	% AGREEMENT (7–9)	FINAL SELECTED ITEMS
**22**	**Dedicated medical staff**			**8**	**93,3**	**8**	**86,7**			**✓**
23	Dedicated pharmacy staff			7	73,3	7	73,3			
**24**	**Dedicated nurse staff**			**8**	**93,3**	**8**	**93,3**			**✓**
**25**	**NICU staff trained in GCP**			**8**	**80,0**	**8**	**86,7**			**✓**
**26**	**Presence “on site” (i.e., institutional) monitoring and research compliance assessment capacity**			**7**	**86,7**	**7**	**80,0**			**✓**
**27**	**Connection(s) to Ethics Committee with paediatric and neonatal expertise (26')**	**26′**	**Connection(s) to Ethics Committee (national, regional, or local) with paediatric and neonatal expertise**	**8**	**93,3**	**8**	**100,0 (≥65%)**	**8**	**93,3**	**✓**
**28**	**Ethics committee national vs local/regional (26')**			**7**	**69,2**					
**29**	**IRB to evaluate the scientific value of the trials**			**8**	**85,7**	**8**	**93,3**			**✓**
**30**	**NICU staff to evaluate local feasibility of the trials**			**8**	**100,0**	**8**	**93,3**			**✓**
**31**	**Possibility for the local investigator to reduce his/her clinical workload to be better able to do the work the trials requires**			**8**	**93,3**	**8**	**100,0**			**✓**
**32**	**Screening of patient to optimize recruitment**			**8**	**86,7**	**8**	**93,3**			**✓**
33	Collaboration with on site biological laboratories	Collaboration with certified biological laboratories		7	71,4	7	66,7			
**34**	**Collaboration with specialized laboratories (PK, PD, PK/PD, etc…)**	**Collaboration with specialized partners (PK, PD, PK/PD, etc…)**		**7**	**78,6**	**7**	**86,7**			**✓**
**35**	**Organization of databases**	**Organization of databases: Data specialist, data entry, and database design/management**		**8**	**100,0**	**8**	**93,3**			**✓**
**36**	**Storage capacity samples (fridges, freezers, etc…)**			**8**	**80,0**	**8**	**93,3**			**✓**
		**36′**	**Access to electronic record**			**7**	**86,7 (≥65%)**	**7**	**80**	**✓**

4. SCIENTIFIC COMPETENCIES AND CAPACITY TO PROVIDE EXPERT ADVICE		3. SCIENTIFIC COMPETENCIES AND EXPERTISES	MEDIAN	% AGREEMENT (7–9)	MEDIAN	% AGREEMENT (7–9)	MEDIAN	% AGREEMENT (7–9)	FINAL SELECTED ITEMS
**49**	**Ability to consult (to collaborate with) experts in the design/conduct of clinical trial**	**Ability to consult (to collaborate with) experts to ensure scientific rigor in study design/conduct of trial**	**8**	**80,0**	**8**	**93,3**			**✓**
**50**	**Ability to consult Regulatory Bodies**		**7**	**86,7**	**8**	**93,3**			**✓**
**51**	**Ability to consult medical expert (cardiologists, surgeons, ophthalmologists, nephrologists, infectiologists, gastroenterologists, endocrinologists, neurologists, etc…)**		**8**	**93,3**	**8**	**93,3**			**✓**
**52**	**Ability to consult neonatal/clinical pharmacology expert**		**8**	**86,7**	**8**	**86,7**			**✓**
**53**	**Ability to consult statistics expert in data-management, pharmaco-statistics and analysis**		**8**	**86,7**	**8**	**93,3**			**✓**
54	NICU team involved as consultants for the industry		6	46,7					
55	Members of the study team are KOLs (Key Opinion Leaders)		6	46,7					
**56**	**Conflicts of interests for members of the team are declared**		**8**	**93,3**	**9**	**100**			**✓**

5. QUALITY MANAGEMENT		4. QUALITY MANAGEMENT		MEDIAN	% AGREEMENT (7–9)	MEDIAN	% AGREEMENT (7–9)	MEDIAN	% AGREEMENT (7–9)	FINAL SELECTED ITEMS
57	Dedicated staff responsible for Quality Assurance of CTs			7	66,7	7	66,7			
58	Regulatory Watch is organized			7	71,4	7	73,3			
**59**	**SOPs for composition of data safety and monitoring boards**			**7,5**	**71,4**	**7,5**	**92,9**			**✓**
**60**	**SOPs on GCP**	**60′**	**SOPs including (adherence to GCP and GLP): General management of the trial; IMP management, Adverse Events Management and Reporting; Ethics…**	**7,5**	**78,6**	**8**	**86,7 (≥65%)**	**8**	**93,3**	**✓**
**61**	**SOPs on Management**			**7**	**57,1**					
**62**	**SOPs on Adverse Events Management**			**8**	**92,9**					
**63**	**SOPs on Ethics**			**8**	**85,7**					
**64**	**Documented Adherence to SOPs**			**8**	**85,7**	**8**	**93,3**			**✓**
**65**	**Regular in-house audit**	**Regular in-house audit if available**		**7**	**64,3**	**7**	**80,0 (≥65%)**	**7**	**86,7**	**✓**
66	Site-inspection audit	Site-inspection if available		7	73,3	7	73,3			
67	Access to patient advocacy group			6	46,7					
**68**	**Certification of the NICU**			**8**	**80,0**	**8**	**93,3**			**✓**
**69**	**Accreditation of the Medical staff**			**8**	**93,3**	**8**	**100,0**			**✓**
70	Scientific watch/diffusion of information			7	64,3					
71	Who reports ADRs?			8	84,6					
**72**	**DSMBs are in place**	**Closed collaboration with DSMBs**		**7**	**84,6**	**7**	**84,6 (≥65%)**	**7**	**92,9**	**✓**

7. PUBLIC INVOLVEMENT		5. PUBLIC INVOLVEMENT		MEDIAN	% AGREEMENT (7–9)	MEDIAN	% AGREEMENT (7–9)	MEDIAN	% AGREEMENT (7–9)	FINAL SELECTED ITEMS
**82**	**Priorisation of needs for clinical trials in children**			**7**	**92,9**	**8**	**93,3**			**✓**
83	Involvement of parents and their organizations in the protocol design			7	53,3					
**81**	**Involvement of patient advocacy group in research activities**	**82′**	**Involvement of patient advocacy group in information leaflets and consent form writing**	**7**	**60,0**	**7,5**	**85,7 (≥65%)**	**7**	**80**	**✓**
**84**	**Information leaflets and consent form writing**			**9**	**100,0**					
**85**	**Information of pregnant women and their partners about research in pregnancy and neonates**			**8**	**86,7**	**8**	**100**			**✓**
**86**	**Enlisting public support through appropriate participation in process/outcomes**			**7**	**66,7**	**7**	**80**			**✓**

The members of the Scientific Committee were selected and contacted by the organisers, and all of them agreed (10/10, 100%) to participate.

Of the twenty panellists appointed by the Scientific Committee to participate to the Delphi rounds, 15 (79%) accepted: 9 were neonatologists, 2 were pharmacologists, 2 were institutional and industrial project managers and 1 was a neonatologist/scientific officer in a regulatory authority. All of them participated in the three rounds of the process.

### First round

**Figure 1:**
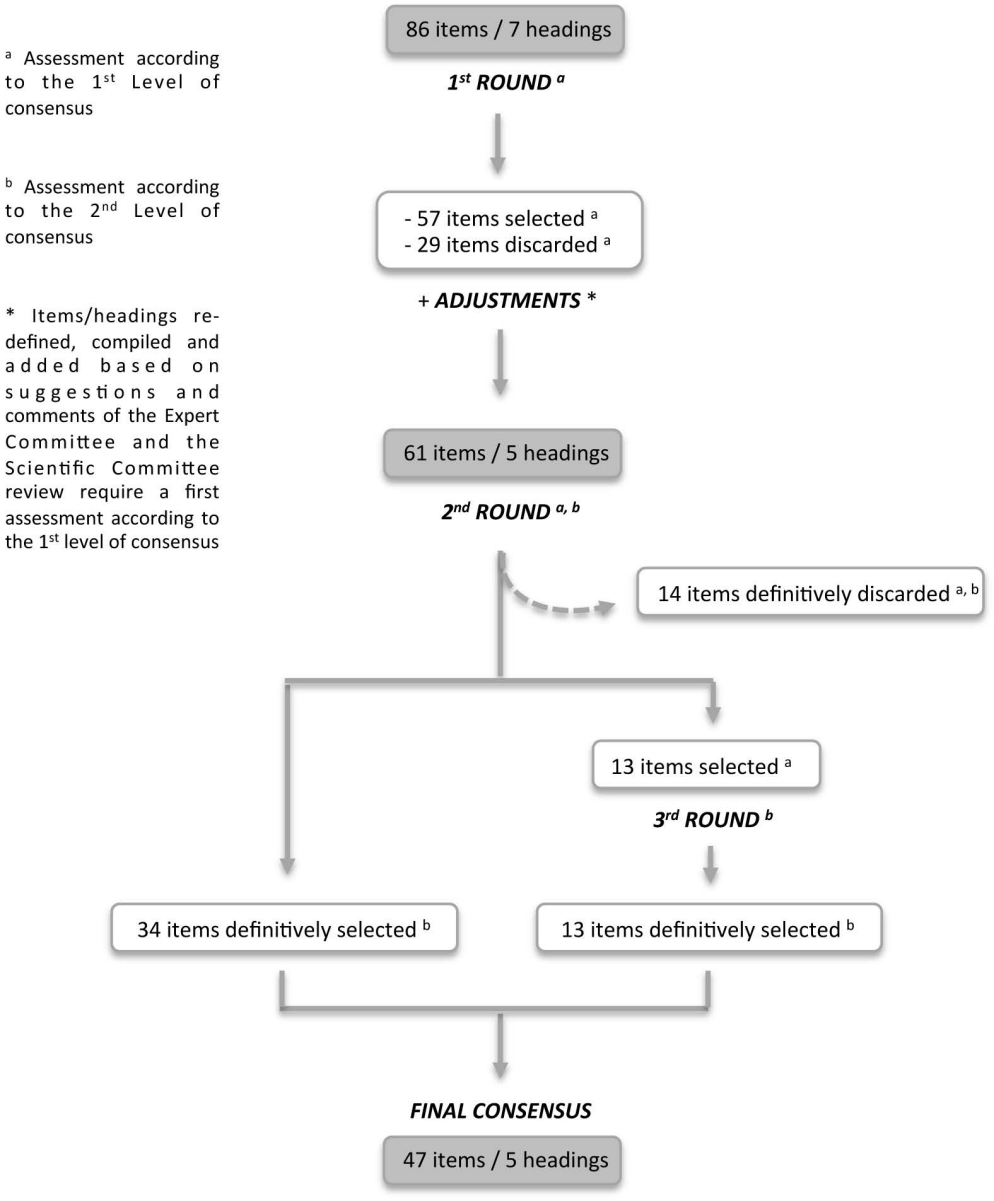
Flowchart of the Delphi process.

The 7 headings comprising a total of 86 initial items tested during the first round are listed in **Table 2** **, column A (** **Figure 1** **)**.

Fifty-seven items reached the first level of consensus (57/86, 66%) (**Table 2** **, column A**), while twenty-nine items were discarded (29/86, 34%): 16 (19%) because of a median rating lower than 7, and 13 (15%) because less than 65% of panellists gave rating in the top tertile [7]–[9].

#### Adjustments at the end of the first round

Adjustments were made prior to the second round, taking into account the suggestions and comments of the Expert Committee and the Scientific Committee review (**Table 2** **, column B**): rearrangements of headings and redefinition of a few items were made, such as H3: “Research Experience” and H6: “Training and educational capacity” that were re-organized and included under the first heading H1: “NICUs Description”; a few items were better defined (H1: i6; and i40; H2: i33 to i35; H3: I49; H4: I65, i66 and i72), while others were compiled into new items (H1: i6′and i75′; H2: i26′; H4: i60′; and H5: i82′); and a new “Access to electronic record” was added (H2: i36′).

After these adjustments, the questionnaire included 61 items under 5 headings (**Table 2** **, column B**) as: from 57 items selected in this first round, 53 items remained; similarly, among the 29 discarded items in the previous round, 7 items were maintained to be re-assessed in the second round; and one additional item was added.

### Second round

At this step of the process, 61 items were therefore evaluated, including modified, retained or additional items (27 items), which were considered as “new items” and were rated for the first time through this second round. Results are presented **Table 2**.

47 items were selected (47/61, 77%). Among them, 34 items were definitively retained based on the second level of consensus, and 13 items required to be re-assessed (second assessment) through a third round (**Figure 1**). The remaining 14 items were, for their part, definitively discarded.

### Third round

The previous 13 items that were re-assessed reached the second level of consensus and were definitively selected.

### Final results

At the end of the process, 47 items were finally selected according to the second level of consensus and divided under 5 headings (**Table 3** and **Figure 1**).

**Table 3:** Final Delphi questionnaire at the end of the 3rd Round.

1. NICUs DESCRIPTION						2. NICU ORGANISATION AND PROCESSES (ABILITY TO PERFORM DRUGS TRIALS)		3. SCIENTIFIC COMPETENCIES AND EXPERTISES		4. QUALITY MANAGEMENT		5. PUBLIC INVOLVEMENT	
1.1. LEVEL OF CARE OF THE NICU		1.2. NICU RESEARCH EXPERIENCE IN DRUG TRIALS: This heading includes items required: 1 - to evaluate once yearly Established units (NICUs with experiences that have already conducted and/or participated in clinical trials). These units will do well on these criteria, 2 - to evaluate Development units (NICUs recently or not yet involved that have the capacity to do research but don't have a track record. NOT REQUIRED TO ENTER THE NETWORK, but for the regular annual assessment		1.3. TRAINING AND EDUCATIONAL CAPACITY OF THE NICU									
**3**	Number of doctors/bed	**37**	Type of clinical trials performed over the last 5 years (phase I/II, phase III/IV, observational studies, PK, PD, PK/PD, efficacy, safety, pharmacoepidemiologic, pharmacovigilance, diagnostic and comportement study, follow-up, etc…)	**73**	Internal training regarding GCPs and human subjects (research ethics) is available	**22**	Dedicated medical staff	**49**	Ability to consult (to collaborate with) experts to ENSURE scientific rigor in study design/conduct of trial	**59**	SOPs for composition of data safety and monitoring boards	**82**	Prioritisation of needs for clinical trials in children
**4**	Number of nurses/bed					**24**	Dedicated nurse staff						
**6**	Ventilation management with a range of respiratory support available (including CPAP and mechanical ventilation)	**38**	Number of completed and ongoing clinical trials over the last 5 years (single centre, national multicenter and international multicenter trials)			**25**	NICU staff trained in GCP	**50**	Ability to consult Regulatory Bodies				
				**74**	Internal training regarding responsibilities of principal investigator and all co-investigators is available			**51**	Ability to consult medical expert (eg cardiologists, surgeons, ophthalmologists, nephrologists, infectivologists, gastroenterologists, endocrinologists, neurologists, etc…)	**60′**	SOPs including (adherence to GCP and GLP): general management of the trial; Adverse Events Management and Reporting; Ethics…		
**12**	Patient follow-up after discharge is available	**39**	Number of investigator (academic) initiated studies performed			**26**	Presence “on site” (i.e., institutional) monitoring and research compliance assessment capacity					**82′**	Involvement of patient advocacy group in information leaflets and consent form writing
		**42**	Numbers of patients enrolled in these trials			**26′**	Connection(s) to Ethics Committee (national, regional, or local) with paediatric and neonatal expertise	**52**	Ability to consult neonatal/clinical pharmacology expert	**64**	Documented Adherence to SOPs		
**14**	Number of infants yearly admitted												
						**29**	IRB to evaluate the scientific value of the trials					**85**	Information of pregnant women and their partners about research in pregnancy and neonates
		**45**	Success rate of real enrollment of the study			**30**	NICU staff to evaluate local feasibility of the trials			**65**	Regular in-house audit if available		
**15**	Number of patients <1500 gram (VLBW) birth weight yearly admitted			**75**	Training courses specific to planned trial	**31**	Possibility for the local investigator to reduce his/her clinical workload to be better able to do the work the trials requires	**53**	Ability to consult statistics expert in data-management, pharmaco-statistics and analyses				
		**46**	Success rate of conduct of the study (adherence to timelines)			**32**	Screening of patient to optimize recruitment			**68**	Certification of the NICU		
						**34**	Collaboration with specialized partners (PK, PD, PK/PD, etc…)					**86**	Enlisting public support through appropriate participation in process/outcomes
**16**	Number of patients <1000 gram (ELBW) birth weight yearly admitted					**35**	Organization of databases: Data specialist, data entry, and database design/management	**56**	Conflicts of interests for members of the team are declared	**69**	Accreditation of the Medical staff		
		**47**	Taking part as a member in neonatal/paediatric networks										
						**36**	Storage capacity samples (fridges, freezers, etc…)			**72**	Closed collaboration with DSMBs		
						**36′**	Access to electronic record						

## Discussion

Drug trials should always be conducted in sites that can guaranty quality, performance and high ethical standards and this is obviously of even greater importance in neonatal drug evaluations. In this context, the present Delphi study was conducted to define criteria that neonatologists should consider to optimise organisation of the NICU, trial management and conduct. Starting from 86 items divided in 7 headings, a consensus among 15 multidisciplinary experts with a wide range of experience in neonatology, experimental or regulatory practices identified 47 items in 5 re-organised headings (**Figure 1**). These criteria should help to set up a network of NICUs specialised in drug evaluation in neonates.

As previously stated, drug research in neonates is difficult and, consequently, the number of neonatal drug trials is limited and sometimes of poor quality [7]. Therefore, collaborative, multicenter and multinational studies are essential to recruit neonates with similar diseases from various regions or countries in order to obtain a sample size of sufficient magnitude and to conduct scientific sound studies. In addition to increasing recruitment capacities, such specialised centers will “combine and share” competences in order to build a network to guaranty trial quality and performances, but also to develop investigator and nurse training. This approach is in agreement with the request of the European Commission and is currently supported by the European Medical Agency that developed the EnprEMA network, “a network of networks” [15].

Most of the paediatric networks that we are aware of are so called “Paediatric improvement networks”, collaborating to reduce the gaps and disparities in health care quality and improve outcomes by accelerating the translation of evidence into practice [16]–[18]. They are mostly organised in paediatric subspecialties such as the Children's Oncology Group (COG) in the USA, the Paediatric Rheumatology INternational Trials Organization (Pinto) and the Paediatric European Network for Treatment of AIDS (Penta) in Europe [15]. Some of them are dedicated to perinatal or neonatal care such as the California Perinatal Quality Care Collaborative (CPQCC) [16], [19] but many other national or regional initiatives do exist [17]. All of them underline the importance of a close link with research although limited networks of NICUs were set up for research purposes. In the USA, the Paediatric Pharmacology Research Units network (PPRU) was a cooperation of clinical centers participating in the cooperative agreement with NICHD and represented academic institutions with experience in multi-center clinical research. They agreed to abide by the study protocols and have comparable staff, facilities, and equipment.

In Europe, the European Neonatal Network (EuroNeoNet) primarily aims to give European neonatologists a tool to perform their own quality assurance and benchmarking. Additional neonatal networks do exist at the national level in most countries, including the German Neonatal Network (GNN), the National Institute for Health Research Medecines for clinical research network (NIHR) in the UK, and the Paediatric Clinical Investigation Centers in France (CIC). However, the criteria that might facilitate initial adhesion and follow-up of the NICUs that are members of a neonatal network are not easily identified. The present Delphi study was conducted in order to combine opinions into group consensus on this topic and is reported here according to published recommendations [20].

The first heading “NICUs description” includes subdivisions focussing on “Level of care, Research experience and Training - Education capacities” with description of the size, organization, and teaching hospital status. All the items related to “highly specialised care technics” (such ECMO, or cooling) were put together as the centers providing them are highly specialised and always identified at the regional or national level. Therefore, the corresponding NICUs will be identified within a network and contacted at the initial step of study feasibility if such technics are required by the protocol.

The term “feasibility” is currently used but may cover different issues: for industry, the question behind feasibility is: do the patients corresponding to the inclusion criteria really exist and in addition, where do we find them? Indeed, when industry-driven, the protocol is developed according to regulatory guidelines for drug evaluation in neonates [3] and follows a Paediatric Investigation Plan that is binding. There are concerns that these may not always feasible in the neonatal population however, as per current experience with TINN (Treat Infections iN Neonates) projects (TINN1 and TINN2) - very few indications of the drug limiting inclusions for a RCT; registry considered as non-informative. In contrast, for clinicians, feasibility primarily means: is this acceptable for the patient and his parents? Do we have time and staff for this? Indeed, analysis of feasibility by a local Scientific Review Board with the medical and nurse staff is essential to evaluate recruitment capacities within predefined calendars and in our experience, the best response may sometimes be NO [21], [22].

Research activities will be reported annually in centers already conducting drug trials, underlining the major importance to be given to performances evaluated in terms of adherence, number of patients included, queries. Continuous re-evaluations of quality and performances are now currently performed in neonatal intensive care and with significant results in terms of long-term quality-adjusted survival [23]. They are also required to evaluate research activities, ensure that all organisational and training efforts are maintained, allowing to reach positive results in terms of recruitment, adherence to timelines, quality and Ethics. Although discussed, additional criteria such as success in grant applications and/or publications were thought to reflect more networking activities than individual NICU activities and performances.

Three headings “NICUs organisation, Scientific competencies, Quality management” include all the items required to guaranty quality and performances of clinical research. Among key items, a clear identification of the research staff, including senior doctors and nurses are key factors to insure quality. The role of nurses in improving neonatal care and outcome has been demonstrated [24]. Similarly, their role in clinical research should be better acknowledged and recognised. As neonates hospitalised in intensive care are the most nurse-intensive patients, conducting clinical drug research requires additional dedicated research nurses. In such context, the required neonate - research/nurse ratio in a given NICU obviously depends upon research workload related to the trials and should also include time for training care nurses, availability for parents' information and all additional tasks related to quality and ethics [25].

Although research in neonates is almost always preceded by research in adults and children, diseases in neonates are different in terms of clinical presentation, evolution and risks. It is therefore essential for researchers and pharmaceutical industry people to get advice from experts in the NICU and its surrounding [26], as involvement in pharmacokinetic and pharmacodynamics drug evaluation, trial designs adapted to low numbers, is expected. The quality management is based on Standard Operating Procedures, i.e documents with detailed instructions, written to describe steps to follow in all activities under defined conditions. They are derived from knowledge of Good Clinical and Laboratory Practices from International Conference on Harmonisation (ICH guideline Q11); and Good Laboratory Practice (GLP) from the Organisation for Economic Cooperation and Development (OECD). Such knowledge is required for all health professionals participating in a clinical trial. In particular, the roles, obligations and responsibilities of the sponsor and all health professionals, respect of ethical standards focussing on parental information and consent are clearly defined. The major “Quality SOPs” available in any research center and that have been used and adapted to the TINN2 multicenter European drug trial in neonates are listed **Table 4**.

**Table 4:** Standard Operating Procedures (SOPs).

**1. Writing, Reviewing & Communication procedures:** Format and style, Document control and version numbering, New SOPs, Review of SOPs, Withdrawal, Training, Communication, Organizing protocol information, etc…	**2. Principles and Procedure of Informed Consent:** Screening, Informing of participants, Obtaining proxy informed consent, Ongoing consent procedure
**3. Amendments to the Protocol and Protocol-Related Documentation:** Identification of need of amendment, Substantial amendments, Non-substantial amendments, Implementation of amendments, Urgent Safety measures	**4. Quality Control and Quality Assurance Procedures:** Standard operating procedures, Trial master file, Protocol, Oversight Committees, Data management and monitoring, Audit and inspection
**5. Trial Pharmacy Procedures - Management of IMP supplies:** Supply and importing of Study Products (IMP and/or Placebo), Packaging and labelling, QP release, Supply chain, Pharmacy procedures, Investigational Drug Accountability Record, Prescription Numbering, Storage requirements, Re-labelling, Product recall	**6. Training and initiation of study centres:** Trial set-up, Delegation of duties and signature log, Site initiation, Site activation, Ongoing training and monitoring
	**7. Preparation and validations of the eCRF**
**8. Management of Essential Documentation:** Trial Master File (TMF), Investigator Site File (ISF), Investigators Brochure, Monitoring plan, Data Management Plan, Statistical Analysis Plan, ISMB charter, Archiving of Essential Documentation, Document storage and access, ISMB charter	**9. Statistical Procedures**
	**10. Pharmacovigilance Procedures:** Reference safety information, Events to be recorded, Pharmacovigilance training, Reporting responsibilities - Principal Investigators, Reporting responsibilities - Sponsor, Development Update Safety Report, Other safety issues
**11. Study closure procedures:** Activities prior to closure, Routine site closure, Early closure	**12. Document Control Procedures:** Generation of new documentation, Version control, Document storage and access, Translation of non-critical controlled documentation, Document revision
**13. Managements of protocol deviations** and serious breach, Deviations pre-identified in monitoring plan, Ad hoc incident reports, etc…	**14. Study reports:** Development update safety report, Annual report to ethics committee, Declaration of end of the trial, End of study report
**15. Laboratory procedures:** Pharmacokinetic samples, Receipt, handling and storage of Microbiology samples, Analysis of Microbiology samples	**16. Trial Auditing and Inspection:** Procedure in case of research fraud and misconduct of the trial, etc…

Among them, “Trial Pharmacy Procedures” including procedures related to “preparation and administration of the IMPs” are essential as dilutions, sometimes multiple, of a concentrated product, low volumes of infusion, potential physico-chemical interactions are key issues [27], [28]. In addition, in this heading, adherence to trial dependent - standard operating procedures should be evaluated by monitors on a regular basis, and documentation and report of all adverse drug reactions.

The last heading is related to “Public involvement”. The philosophy of “Family-centered care” referring to a partnership between parents and the medical staff [29] focuses on involving them in all ethical and medical decisions related to their newborn's care [30]. Similarly, decisions regarding neonatal research enrollment need to be made conjointly with parents and health care professionals and many studies are now available to improve the consent process [31]–[33]. Researchers are also concerned by involving parents in an earlier phase, in order to help drafting the information and consent documents and make them more accessible to parents, as reflected in the selected items kept in the heading. One item “Involvement of parents and their representatives or organisations in trial design” was not selected, probably because “Trial design” refers to specific methodologies to optimize drug trials taking into account neonatal specificities. In contrast, trials dealing with safety evaluation or long-term follow up are to be discussed extensively with parents in order to evaluate their view on how the child and the whole family will be involve, on how to face open questions on outcome and potential sequelae or neurodevelopmental disorders.

In conclusion, this is to our knowledge, the first consensus obtained by experts and aiming to list the items that should be considered to organize a neonatal network of specialised intensive care units dedicated to drug evaluation. Some of these items may not be required in all units but should be identified to optimize trial design, conduct highly specialised evaluations and train health professionals to trial conduct for the benefit of neonates and their parents.
